# Supplementary material for: Vestigial singlet pairing in a fluctuating magnetic triplet superconductor and its implications for graphene superlattices
Source: Nat Commun. 2024 Feb 24;15:1713. doi: 10.1038/s41467-024-45950-4 (PMC10894192; doi:10.1038/s41467-024-45950-4)
Supplement: Supplementary file 1 — Supplementary Information [file 41467_2024_45950_MOESM1_ESM.pdf]

Supplementary Information for "Vestigial singlet pairing in a fluctuating magnetic triplet superconductor and its implications for graphene superlattices"

Prathyush P. Poduval[1,2] and Mathias S. Scheurer[3,4]

[1] *Donald Bren School of Information and Computer Sciences, University of California, Irvine, CA 92697, USA*
[2] *Condensed Matter Theory Center, Department of Physics, University of Maryland, College Park, MD 20742, USA*
[3] *Institute for Theoretical Physics III, University of Stuttgart, 70550 Stuttgart, Germany*
[4] *Institute for Theoretical Physics, University of Innsbruck, Innsbruck A-6020, Austria*

Appendix A: Self-consistent equations in special limits

In this appendix, we complement the previous analysis by studying two simple limits of the model for phase (B)—mean-field theory and the limit of zero energy-momentum transfer of the bosons. This allows us to study possible non-perturbative solutions systematically. In both cases, we find that the soft gap behavior obtained within perturbation theory is also found in these descriptions as long as T is large enough/the coupling constants, λ or ϕ_0, are small enough.

1. Mean-field Theory

In this section, we consider the effective interaction contributed by the $\mathcal{S}_2$ part of the action between the electrons at time $t = 0$, in the limit where we replace the q integral with the corresponding value of the integrand at $q = 0$, and then perform a mean-field decomposition of the interaction. Defining the Bogoliubov-de Gennes basis as before, $\xi_k = \begin{pmatrix} c_{k,+} & is_y c^\dagger_{-k,-} \end{pmatrix}^T$, with Pauli matrix γ_i acting on it, and $\tilde{\phi}_0 = \phi_0 \lambda^2 r_N / v_N^2$ the corresponding interaction potential is given by

$$V = -\frac{1}{2}\frac{1}{\chi_d^{-1}\chi_N^{-1} - |\phi_0|^2}\left(\tilde{\phi}_0 \mathbf{S}_{q=0}\cdot \mathbf{D}^\dagger_{q=0} + \tilde{\phi}_0^* \mathbf{D}_{q=0}\cdot \mathbf{S}_{-q=0}\right)|_{q=0} \tag{A1}$$

$$= -\frac{1}{2}\frac{1}{r_N r_d - |\phi_0|^2}\int_{\boldsymbol{k}_1,\boldsymbol{k}_2}\left[-\tilde{\phi}_0\left(c^\dagger_{\boldsymbol{k}_1}\mathbf{s}\tau_z c_{\boldsymbol{k}_1}\right)\cdot\left(c_{\boldsymbol{k}_2}\mathbf{s} i s_y \tau_y c_{-\boldsymbol{k}_2}\right) + h.c\right] \tag{A2}$$

$$= -\frac{1}{r_N r_d - |\phi_0|^2}\int_{\boldsymbol{k}_1,\boldsymbol{k}_2}\left[\tilde{\phi}_0\left(\xi^\dagger_{\boldsymbol{k}_1}\mathbf{s}\gamma_z \xi_{\boldsymbol{k}_1}\right)\cdot\left(\xi^\dagger_{\boldsymbol{k}_2}\mathbf{s} i\gamma_- \xi_{\boldsymbol{k}_2}\right) + h.c\right], \tag{A3}$$

while the free Hamiltonian is given by

$$H_0 = \int_{\boldsymbol{k}} \xi^\dagger_{\boldsymbol{k}}\epsilon_{\boldsymbol{k}}\gamma_z \xi_{\boldsymbol{k}}. \tag{A4}$$

We consider only the effective Hamiltonian at time $t = 0$, which is why there are no Matsuabra indices.

We perform a Hartree-Fock decomposition of V, which gives us

$$V = \frac{1}{r_N r_d - |\phi_0|^2}\int_{\boldsymbol{k}_1,\boldsymbol{k}_2}\left[\tilde{\phi}_0\left(\xi^\dagger_{\boldsymbol{k}_1}\mathbf{s}\gamma_z \xi_{\boldsymbol{k}_1}\right)\cdot\left(\xi^\dagger_{\boldsymbol{k}_2}\mathbf{s} i\gamma_- \xi_{\boldsymbol{k}_2}\right) + h.c\right] \tag{A5}$$

$$\rightarrow \frac{c}{2}\int_{\boldsymbol{k}} \xi^\dagger_{\boldsymbol{k}}\left(\gamma_y C_{\boldsymbol{k}}\gamma_z + \gamma_z C_{\boldsymbol{k}}\gamma_y\right)\xi_{\boldsymbol{k}}, \tag{A6}$$

where $C_{\boldsymbol{k}} = -\langle \xi_{\boldsymbol{k}}\xi^\dagger_{\boldsymbol{k}}\rangle$, $c = 6\frac{\tilde{\phi}_0}{r_N r_d - \phi_0^2}$, choosing a gauge with real ϕ_0; further take ϕ_0 to be positive such that $c > 0$. Note that this correlator is related to the Green's function G by $C_{\boldsymbol{k}} = T\sum_{i\omega_n} G(k)$. Note that all the Hartree terms

vanish since we do not allow for spontaneous breaking of spin-rotation invariance (recall we study finite T in 2D). The effective 2−particle Hamiltonian is given by

$$H = \int_{\boldsymbol{k}} \xi_{\boldsymbol{k}}^\dagger \left(\epsilon_{\boldsymbol{k}} \gamma_z + \frac{c}{2} \gamma_y C_{\boldsymbol{k}} \gamma_z + \frac{c}{2} \gamma_z C_{\boldsymbol{k}} \gamma_y \right) \xi_{\boldsymbol{k}} \tag{A7}$$

$$= \int_{\boldsymbol{k}} \xi_{\boldsymbol{k}}^\dagger \left[\tilde{\epsilon}_{\boldsymbol{k}} \gamma_z + \tilde{\Delta}_{\boldsymbol{k}} \gamma_y \right] \xi_{\boldsymbol{k}} \tag{A8}$$

where $\tilde{\epsilon}_{\boldsymbol{k}}, \tilde{\Delta}_{\boldsymbol{k}}$ are the self consistent band structure and gap. Making connection with the diagrammatic self consistency relationship to be discussed below, we can foresee that the resulting self consistent equation we get will be the same as (A18) but with $\tilde{\epsilon}, \tilde{\Delta}$ replaced with the corresponding $i\omega_n$ averaged value, and the whole equation itself will be $i\omega_n$ averaged.

The correlators in terms of $\tilde{\epsilon}, \tilde{\Delta}$ are given by

$$C_{\boldsymbol{k}} = T \sum_{i\omega_n} \frac{1}{i\omega_n - \left[\tilde{\epsilon}_{\boldsymbol{k}} \gamma_z + \tilde{\Delta}_{\boldsymbol{k}} \gamma_y \right]} = \frac{n_f(E_{\boldsymbol{k}}) - n_f(-E_{\boldsymbol{k}})}{2E_{\boldsymbol{k}}} \left[\tilde{\epsilon}_{\boldsymbol{k}} \gamma_z + \tilde{\Delta}_{\boldsymbol{k}} \gamma_y \right], \tag{A9}$$

where $E_{\boldsymbol{k}} = \sqrt{\tilde{\epsilon}_{\boldsymbol{k}}^2 + \tilde{\Delta}_{\boldsymbol{k}}^2} > 0$. Thus, using (A7), the self consistency equations become

$$\tilde{\epsilon}_{\boldsymbol{k}} = \epsilon_{\boldsymbol{k}} + c \tilde{\Delta}_{\boldsymbol{k}} \frac{n_f(E_{\boldsymbol{k}}) - n_f(-E_{\boldsymbol{k}})}{2E_{\boldsymbol{k}}} \tag{A10}$$

$$\tilde{\Delta}_{\boldsymbol{k}} = c \tilde{\epsilon}_{\boldsymbol{k}} \frac{n_f(E_{\boldsymbol{k}}) - n_f(-E_{\boldsymbol{k}})}{2E_{\boldsymbol{k}}}. \tag{A11}$$

Let us define $\beta_{\boldsymbol{k}} = c \frac{n_f(-E_{\boldsymbol{k}}) - n_f(E_{\boldsymbol{k}})}{2E_{\boldsymbol{k}}} = c \frac{\tanh\left(\frac{E_{\boldsymbol{k}}}{2T}\right)}{2E_{\boldsymbol{k}}}$ and first assume $\beta_{\boldsymbol{k}} < 1$, which always holds as long as $T > c/4$. The self consistency equations can then be rearranged as

$$\tilde{\epsilon}_{\boldsymbol{k}} = \frac{1}{1 - \beta_{\boldsymbol{k}}^2} \epsilon_{\boldsymbol{k}} \tag{A12a}$$

$$\tilde{\Delta}_{\boldsymbol{k}} = \frac{-\beta_{\boldsymbol{k}}}{1 - \beta_{\boldsymbol{k}}^2} \epsilon_{\boldsymbol{k}}. \tag{A12b}$$

Using this, we find $E_{\boldsymbol{k}} = \frac{\sqrt{1+\beta_{\boldsymbol{k}}^2}}{1-\beta_{\boldsymbol{k}}^2} \epsilon_{\boldsymbol{k}}$. Note, however, that $\beta_{\boldsymbol{k}}$ also depends on $E_{\boldsymbol{k}}$ and, thus, this relation should be thought of as a self consistency equation, to be solved for $\beta_{\boldsymbol{k}}$ or $E_{\boldsymbol{k}}$.

Equations (A12) allow to derive asymptotic relations. In the limit $\epsilon_{\boldsymbol{k}} \to 0$, we then have $E_{\boldsymbol{k}} \to 0$ and $\beta_{\boldsymbol{k}} \to \frac{c}{4T}$, ensuring the self-consistent solutions are well controlled in the $\epsilon_{\boldsymbol{k}} \to 0$ regime that we are interested in. Near $\epsilon_{\boldsymbol{k}} = 0$ and for large $T \gg c$ ($\beta_{\boldsymbol{k}} \ll 1$), the renormalized spectrum is given by $E_{\boldsymbol{k}} = \frac{\sqrt{1+\beta_{\boldsymbol{k}}^2}}{1-\beta_{\boldsymbol{k}}^2} \epsilon_{\boldsymbol{k}} \simeq \sqrt{1 + 3\beta_{\boldsymbol{k}}^2} \epsilon_{\boldsymbol{k}} \simeq \sqrt{1 + \frac{3c^2}{16T^2}} \epsilon_{\boldsymbol{k}}$. The suppression of DOS is now given by

$$\frac{\rho_F(\phi_0)}{\rho_F(\phi_0 = 0)} = \frac{1}{\sqrt{1 + \alpha'^2}}, \quad \alpha' = \frac{3\sqrt{3}\phi_0 \lambda^2 r_N}{2v_N^2 T(r_d r_N - \phi_0^2)},$$

which is of the same form as Eq. (4), found through the perturbative calculation presented in the main text and derived in Sec. D.

When $T/c = 1/4$, we have $\beta_{\boldsymbol{k}}^2 = 1$ for $\epsilon_{\boldsymbol{k}} \to 0$, and Eq. (A12) are not valid. At this point, the self consistent solutions open up a gap in $E_{\boldsymbol{k}}$ when $\epsilon_{\boldsymbol{k}} = 0$. This gap follows by solving the equation $\beta_{\boldsymbol{k}}^2 = 1$. When $\epsilon_{\boldsymbol{k}} = 0$ and $\beta_{\boldsymbol{k}} = 1$, we also have $\tilde{\epsilon}_{\boldsymbol{k}} = -\tilde{\Delta}_{\boldsymbol{k}}$ [see Eq. (A11)] which gives $E_{\boldsymbol{k}} = \sqrt{2}\tilde{\epsilon}_{\boldsymbol{k}}$. For T/c approaching 1/4 from below, we find that $\beta_{\boldsymbol{k}} \simeq \frac{c}{4T}\left(1 - \frac{1}{12}\frac{E_{\boldsymbol{k}}^2}{T^2}\right)$. Thus the condition that $\beta_{\boldsymbol{k}}^2 = 1$ gives us $E_{\boldsymbol{k}} = \sqrt{12}T\sqrt{1 - \frac{4T}{c}}$.

To summarize, for $T > c/4$, self consistent energy and gap $(\tilde{\epsilon}, \tilde{\Delta})$ are proportional to ϵ. As T approaches $c/4$ from above, the slope of proportionality approaches ∞ at $\epsilon = 0$, and becomes non-analytic at $T = c/4$. Going below $T = c/4$, this non-analyticity at $\epsilon = 0$ turns into a discontinuity at $\epsilon = 0$, with the self consistent solutions developing a finite gap. The value of this gap at $T = 0$ is given as $|\tilde{\Delta}| = |\tilde{\epsilon}| = \frac{|c|}{2\sqrt{2}}$. Figure 1 illustrates the behavior obtained by numerical solution of the self-consistency equations.

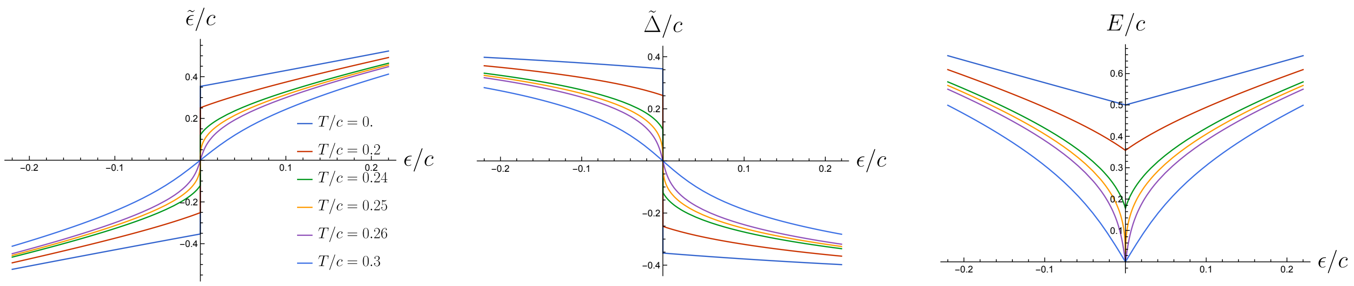

FIG. 1: The self consistent solution for $\tilde{\epsilon}_{\boldsymbol{k}}, \tilde{\Delta}_{\boldsymbol{k}}$ and $E_{\boldsymbol{k}}$ as a function of $\epsilon_{\boldsymbol{k}}$ for various temperatures. At $T/c = 1/4$, the self-consistent solutions become non-analytic having an infinite slope at $\epsilon_{\boldsymbol{k}} = 0$, and a gap opens up as the temperature decreases. There is a discontinuity in $\tilde{\epsilon}_{\boldsymbol{k}}, \tilde{\Delta}_{\boldsymbol{k}}$ at $\epsilon_{\boldsymbol{k}} = 0$, where the gap value has different signs for $\epsilon_{\boldsymbol{k}} \to 0^-, 0^+$.

2. Zero energy-momentum transfer

In this section, we consider the limit where the bosonic fields $\boldsymbol{N}, \boldsymbol{d}$ do not transfer any momentum or Matsubara frequency in the interaction ($q = 0$ in $\mathcal{S}_c$). Additionally, we consider only the effect of $\mathcal{S}_2$ on the self energy to study the effect of the anomalous contribution. In this limit, we would like to analyze the self consistent solution of the Green's function up to all orders in λ within the large-N theory of the main text. The ansatz of the full Green's function is given by $G^{-1} = i\omega_n - \tilde{\epsilon}_k\gamma_z - \tilde{\Delta}_k\gamma_y$, since Σ_3 renormalizes only the anomalous term $\tilde{\Delta}_k$ and the spectrum $\tilde{\epsilon}_k$. We have

$$G = \frac{i\omega_n + \tilde{\epsilon}_k\gamma_z + \tilde{\Delta}_k\gamma_y}{(i\omega_n)^2 - \tilde{\epsilon}_k^2 - \tilde{\Delta}_k^2}. \tag{A13}$$

Thus the self-consistent analogue of Σ_3 in Eq. (3) becomes (where we have replaced the integration over q by the $q = 0$ value of the integrand, and $\tilde{\phi}_0 = \phi_0\lambda^2 r_N/v_N^2$)

$$\Sigma_3 = 6T\frac{\tilde{\phi}_0}{r_N r_d - \phi_0^2}\frac{\tilde{\epsilon}_k\gamma_y + \tilde{\Delta}_k\gamma_z}{(i\omega_n)^2 - \tilde{\epsilon}_k^2 - \tilde{\Delta}_k^2}. \tag{A14}$$

From the self-energy equation we get

$$G^{-1} = G_0^{-1} - \Sigma_3 \tag{A15}$$

$$i\omega_n - \tilde{\epsilon}_k\gamma_z - \tilde{\Delta}_k\gamma_y = i\omega_n - \epsilon_{\boldsymbol{k}}\gamma_z - 6T\frac{\tilde{\phi}_0}{r_N r_d - \phi_0^2}\frac{\tilde{\epsilon}_k\gamma_y + \tilde{\Delta}_k\gamma_z}{(i\omega_n)^2 - \tilde{\epsilon}_k^2 - \tilde{\Delta}_k^2} \tag{A16}$$

$$\tilde{\epsilon}_k = \epsilon_{\boldsymbol{k}} + Tc\frac{\tilde{\Delta}_k}{(i\omega_n)^2 - \tilde{\epsilon}_k^2 - \tilde{\Delta}_k^2} \tag{A17}$$

$$\tilde{\Delta}_k = Tc\frac{\tilde{\epsilon}_k}{(i\omega_n)^2 - \tilde{\epsilon}_k^2 - \tilde{\Delta}_k^2}, \tag{A18}$$

where $c = \frac{6\tilde{\phi}_0}{r_N r_d - \phi_0^2}$. Right at the Fermi surface, $\epsilon_{\boldsymbol{k}} = 0$, the self consistency equations reduce to

$$\tilde{\epsilon}_k = Tc\frac{\tilde{\Delta}_k}{(i\omega_n)^2 - \tilde{\epsilon}_k^2 - \tilde{\Delta}_k^2} \tag{A19}$$

$$\tilde{\Delta}_k = Tc\frac{\tilde{\epsilon}_k}{(i\omega_n)^2 - \tilde{\epsilon}_k^2 - \tilde{\Delta}_k^2} \tag{A20}$$

$$\Longrightarrow \tilde{\epsilon}_k = T^2c^2\frac{\tilde{\epsilon}_k}{(\omega_n^2 + \tilde{\epsilon}_k^2 + \tilde{\Delta}_k^2)^2} \tag{A21}$$

There are two possible solutions to Eqs. (A19) and (A20). The first is $\tilde{\epsilon}_k = \tilde{\Delta}_k = 0$; this is exactly what we find within perturbation theory. For a solution with $\tilde{\epsilon}_k \neq 0$ to exist, it must hold (assuming $\tilde{\epsilon}_k$, $\tilde{\Delta}_k \in \mathbb{R}$ as expected in the

gauge that we use)

$$1 = T^2 \frac{c^2}{(\omega_n^2 + \tilde{\epsilon}_k^2 + \tilde{\Delta}_k^2)^2} < T^2 \frac{c^2}{\pi^4 T^4} \tag{A22}$$

Thus, a non-zero solution only exists if $T < c/\pi^2 \sim c/9$. As compared to Hartree-Fock, the critical temperature for a non-perturbative solution is lower.

Appendix B: Evaluation of the self-energies at leading order

In this section, we show the evaluation of the self energies up to first order in perturbation theory. We first evaluate the anomalous part of the self energy, Σ_3 in Fig. 2(b), which is contributed by the anomalous term of the action given by

$$\mathcal{S}_2 = -\frac{1}{2} \int_q \frac{\lambda^2}{\chi_d^{-1}\chi_N^{-1} - |\phi_0|^2} \left(\phi_0 \mathbf{S}_q \cdot \mathbf{D}_q^\dagger + \phi_0^* \mathbf{D}_q \cdot \mathbf{S}_{-q} \right). \tag{B1}$$

In the following, we work in the $\begin{pmatrix} c_{q,+} & i s_y c_{-q,-}^\dagger \end{pmatrix}^T$ Bogoliubov-de Gennes basis, with the Pauli matrices γ_i acting on it. The free Green's function then reads as $G_0^{-1}(k) = i\omega - \epsilon_{\boldsymbol{k}} \gamma_z$. Choosing ϕ_0 to be real, we have

$$\Sigma_3 = 3 \int_q \frac{\phi_0 \lambda^2}{M_q} \left(\gamma_y G_{0,k+q} \gamma_z + \gamma_z G_{0,k+q} \gamma_y \right) = 6 \int_q \frac{\phi_0 \lambda^2}{M_q} \frac{\epsilon_{\boldsymbol{k}+\boldsymbol{q}}}{(i\omega + i\Omega)^2 - \epsilon_{\boldsymbol{k}+\boldsymbol{q}}^2} \gamma_y, \tag{B2}$$

where

$$M_q = \chi_N^{-1}\chi_d^{-1} - \phi_0^2 = \left(-(i\Omega)^2 + r_N + v_N^2 \boldsymbol{q}^2 \right) \left(-(i\Omega)^2 + r_d + v_d^2 \boldsymbol{q}^2 \right) - \phi_0^2 \tag{B3}$$

$$= ((i\Omega)^2 - E_+^2)((i\Omega)^2 - E_-^2), \tag{B4}$$

with $E_\pm^2 = \frac{g_d + g_N \pm \sqrt{(g_d - g_N)^2 + 4\phi_0^2}}{2}$, and $g_\mu = r_\mu + v_\mu^2 \boldsymbol{q}^2$. Thus,

$$\Sigma_3 = 6\phi_0 \lambda^2 \int_{\boldsymbol{q}} T \sum_{i\Omega \in \text{Bosonic}} \frac{1}{\left((i\Omega)^2 - E_+(\boldsymbol{q})^2 \right) \left((i\Omega)^2 - E_-(\boldsymbol{q})^2 \right)} \frac{\epsilon_{\boldsymbol{k}+\boldsymbol{q}}}{(i\omega + i\Omega)^2 - \epsilon_{\boldsymbol{k}+\boldsymbol{q}}^2} \gamma_y. \tag{B5}$$

The Matsubara sum can be evaluated using

$$f(i\omega, \epsilon) = T \sum_{i\Omega} \frac{1}{((i\Omega)^2 - E_+^2)((i\Omega)^2 - E_-^2)} \frac{1}{i\omega + i\Omega - \epsilon} \tag{B6}$$

$$= \frac{1}{2} \frac{1}{E_+^2 - E_-^2} \left(\frac{1}{E_+} \left(K(i\omega, \epsilon, E_+) - K(i\omega, \epsilon, -E_+) \right) - \frac{1}{E_-} \left(K(i\omega, \epsilon, E_-) - K(i\omega, \epsilon, -E_-) \right) \right), \tag{B7}$$

$$K(i\omega, \epsilon, E) = \frac{n_f(\epsilon) + n_B(-E)}{E + \epsilon - i\omega}, \tag{B8}$$

where $n_{f/B}(\epsilon) = \frac{1}{e^{\beta\epsilon} \pm 1}$. Thus we get,

$$\Sigma_3(k) = 3\phi_0 \lambda^2 \int_{\boldsymbol{q}} \left(f(i\omega, \epsilon_{\boldsymbol{k}+\boldsymbol{q}}) - f(i\omega, -\epsilon_{\boldsymbol{k}+\boldsymbol{q}}) \right) \gamma_y, \tag{B9}$$

where we performed a partial fraction decomposition of $\frac{2\epsilon_{\boldsymbol{k}+\boldsymbol{q}}}{(i\omega + i\Omega)^2 - \epsilon_{\boldsymbol{k}+\boldsymbol{q}}^2} = \frac{1}{i\omega + i\Omega - \epsilon_{\boldsymbol{k}+\boldsymbol{q}}} - \frac{1}{i\omega + i\Omega + \epsilon_{\boldsymbol{k}+\boldsymbol{q}}}$ to arrive at the expression.

The normal part of the self energy, $\Sigma_{1,2}$ in Fig. 2(b), is contributed by the following term of the action

$$\mathcal{S}_1 = -\int_q \frac{\lambda^2}{\chi_d^{-1}\chi_N^{-1} - |\phi_0|^2} \left(\frac{\chi_d^{-1}}{4} \mathbf{S}_q \cdot \mathbf{S}_{-q} + \chi_N^{-1} \mathbf{D}_q \cdot \mathbf{D}_q^\dagger \right). \tag{B10}$$

Defining $\gamma_\pm = \frac{1}{2}(\gamma_x \pm i\gamma_y)$, the corresponding contribution to the self energy is given by

$$\Sigma_1 + \Sigma_2 = \int_q \frac{\lambda^2}{M_q}\left[6\frac{\chi_d^{-1}(q)}{4}\gamma_z G_{0,k+q}\gamma_z + 12\chi_N^{-1}(q)\left(\gamma_+ G_{0,k+q}\gamma_- + \gamma_- G_{0,k+q}\gamma_+\right)\right] \tag{B11}$$

$$= \int_{\boldsymbol{q}} T \sum_{i\Omega\in\text{Bosonic}} \frac{\lambda^2}{M_q}\frac{1}{(i\omega+i\Omega)^2 - \epsilon_{\boldsymbol{k}+\boldsymbol{q}}^2}\left[\frac{2}{3}(g_d - (i\Omega)^2)\left(i\omega + i\Omega + \epsilon_{\boldsymbol{k}+\boldsymbol{q}}\gamma_z\right) + 12(g_N - (i\Omega)^2)(i\omega + i\Omega - \epsilon_{\boldsymbol{k}+\boldsymbol{q}}\gamma_z)\right]. \tag{B12}$$

Note that $\gamma_z G_0 \gamma_z = G_0 = i\omega - \epsilon\gamma_z$, while $\gamma_- G_0 \gamma_+ + \gamma_+ G_0 \gamma_- = i\omega + \epsilon\gamma_z$. As a result, if we consider the self energies as function of $i\omega$ and $\epsilon_{\boldsymbol{k}+\boldsymbol{q}}$, we find that $\Sigma_1 \sim \lambda^2 \int_q \frac{3\chi_d^{-1}(q)}{2M_q} G_0(i\omega, \epsilon_{\boldsymbol{k}+\boldsymbol{q}})$ while $\Sigma_2 \sim \lambda^2 \int_q \frac{12\chi_N^{-1}(q)}{M_q} G_0(i\omega, -\epsilon_{\boldsymbol{k}+\boldsymbol{q}})$. This allows us to argue the effect of Σ_2 pushing high energy states towards the vicinity of $\omega = 0$, while Σ_1 pushes states away from $\omega = 0$.

To perform the Matsubara sums, we define

$$h(i\omega, \epsilon, g) = T\sum_{i\Omega} \frac{-(i\Omega)^2 + g}{((i\Omega)^2 - E_+^2)((i\Omega)^2 - E_-^2)}\frac{1}{i\omega + i\Omega - \epsilon} \tag{B13}$$

$$= \frac{1}{2}\frac{1}{E_+^2 - E_-^2}\left(\frac{E_+^2 - g}{E_+}\left(K(i\omega,\epsilon,E_+) - K(i\omega,\epsilon,-E_+)\right) - \frac{E_-^2 - g}{E_-}\left(K(i\omega,\epsilon,E_-) - K(i\omega,\epsilon,-E_-)\right)\right), \tag{B14}$$

with $K(i\omega, \epsilon, E)$ as defined in (B8). In terms of these functions, the self energy is given by

$$\Sigma_1 = \lambda^2 \int_{\boldsymbol{q}} \frac{1}{3}\left[\left(h(i\omega, \epsilon_{\boldsymbol{k}+\boldsymbol{q}}, g_d) + h(i\omega, -\epsilon_{\boldsymbol{k}+\boldsymbol{q}}, g_d)\right) + \left(h(i\omega, \epsilon_{\boldsymbol{k}+\boldsymbol{q}}, g_d) - h(i\omega, -\epsilon_{\boldsymbol{k}+\boldsymbol{q}}, g_d)\right)\gamma_z\right], \tag{B15}$$

$$\Sigma_2 = \lambda^2 \int_{\boldsymbol{q}} 6\left[\left(h(i\omega, \epsilon_{\boldsymbol{k}+\boldsymbol{q}}, g_N) + h(i\omega, -\epsilon_{\boldsymbol{k}+\boldsymbol{q}}, g_N)\right) - \left(h(i\omega, \epsilon_{\boldsymbol{k}+\boldsymbol{q}}, g_N) - h(i\omega, -\epsilon_{\boldsymbol{k}+\boldsymbol{q}}, g_N)\right)\gamma_z\right]. \tag{B16}$$

We can expand the total self energy $\Sigma = \Sigma_1 + \Sigma_2 + \Sigma_3$ in terms of Pauli matrices in Nambu space,

$$\Sigma(k) = \Sigma_{Id}(k) + \Sigma_z(k)\gamma_z + \Sigma_{\gamma_y}(k)\gamma_y, \tag{B17}$$

where

$$\Sigma_{Id}(k) = \lambda^2 \int_{\boldsymbol{q}} \left[\frac{1}{3}\left(h(i\omega, \epsilon_{\boldsymbol{k}+\boldsymbol{q}}, g_d) + h(i\omega, -\epsilon_{\boldsymbol{k}+\boldsymbol{q}}, g_d)\right) + 6\left(h(i\omega, \epsilon_{\boldsymbol{k}+\boldsymbol{q}}, g_N) + h(i\omega, -\epsilon_{\boldsymbol{k}+\boldsymbol{q}}, g_N)\right)\right], \tag{B18}$$

$$\Sigma_z(k) = \lambda^2 \int_{\boldsymbol{q}} \left[\frac{1}{3}\left(h(i\omega, \epsilon_{\boldsymbol{k}+\boldsymbol{q}}, g_d) - h(i\omega, -\epsilon_{\boldsymbol{k}+\boldsymbol{q}}, g_d)\right) - 6\left(h(i\omega, \epsilon_{\boldsymbol{k}+\boldsymbol{q}}, g_N) - h(i\omega, -\epsilon_{\boldsymbol{k}+\boldsymbol{q}}, g_N)\right)\right], \tag{B19}$$

$$\Sigma_{\gamma_y}(k) = 3\phi_0\lambda^2 \int_{\boldsymbol{q}} \left[f(i\omega, \epsilon_{\boldsymbol{k}+\boldsymbol{q}}) - f(i\omega, -\epsilon_{\boldsymbol{k}+\boldsymbol{q}})\right]. \tag{B20}$$

Appendix C: Higher-order corrections to electronic Green's function

In this section, we show comparisons between the first order perturbative solution and the full self consistent solution to the fermionic Green's function. We define the corrected Green's function to be $G(i\omega, \boldsymbol{k}) = i\omega Z_{\boldsymbol{k}}(i\omega) - \varepsilon_{\boldsymbol{k}}(i\omega)\gamma_z + \Delta_{\boldsymbol{k}}(i\omega)\gamma_y$. In practice, we find that $Z_{\boldsymbol{k}}(i\omega) \simeq 1$, so we focus on $\varepsilon_{\boldsymbol{k}}(i\omega)$ and $\Delta_{\boldsymbol{k}}(i\omega)$ in the following.

In Fig. 2, we show a comparison of the first order result for $\varepsilon_{\boldsymbol{k}}(i\omega_n)$ and $\Delta_{\boldsymbol{k}}(i\omega_n)$ after including the evaluation of the Σ_3 term of the self energy [last diagram in Fig. 2(b)] and the full self consistent solution to the self energy in Matsubara space [obtained by summing up the diagrams in Fig. 2(a) corresponding to Σ_3] at fixed $\boldsymbol{k}$. We find that for small values up to $\phi_0 \sim 0.6 r_N$, the first order and self consistent solutions differ little. In first order, $\varepsilon_{\boldsymbol{k}}(i\omega_n)$ does not get renormalized since Σ_3 acquires a γ_z term only if the Green's function has a γ_y term. Such a γ_y term does not exist in the normal state about which we perform perturbation theory. As ϕ_0 increases, we find that the self consistent solution is lower in magnitude that the first order solution.

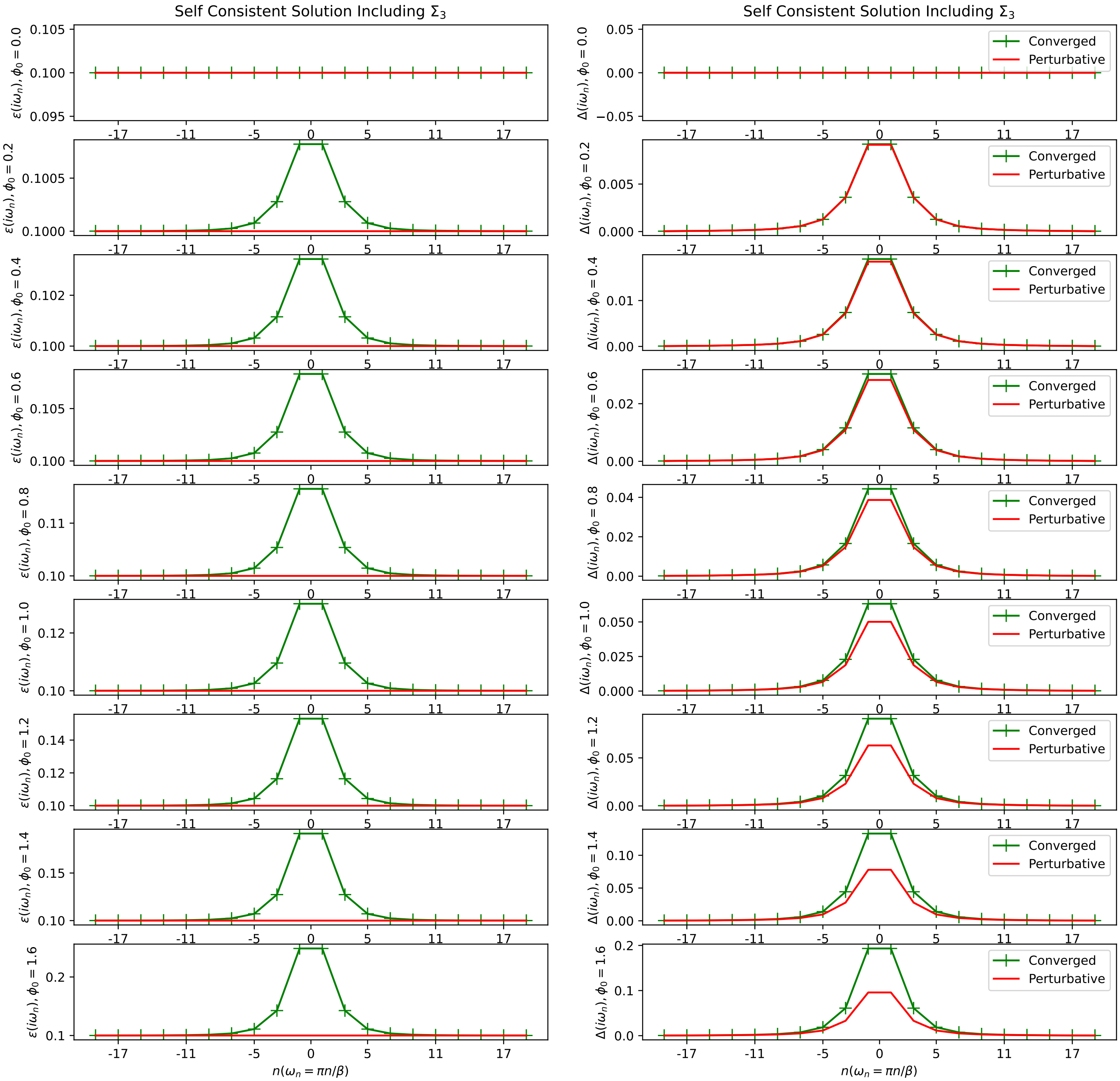

FIG. 2: The first order solution to $\varepsilon(i\omega), \Delta(i\omega)$ (red) and the self consistent solution (green) for the self energy in Matsubara space. Note the offset by 0.1 in the y axis in the left column. We chose $\epsilon_{\boldsymbol{k}} = 0.1, r_d = 9, r_N = 1, T = \frac{1}{\beta} = 0.2, \lambda = 1$ and measured all energies in units of $\sqrt{r_N}$.

In Fig. 3, we show the corrections in $\varepsilon(i\omega_n)$ after including the effects of Σ_1 (left column) and Σ_2 (right column). As expected and argued in the main text, we find that Σ_1 and Σ_2 have qualitatively the opposite effects on the renormalization of $\varepsilon(i\omega_n)$. In both the cases, we find that the magnitude of the self consistent solution is higher than the perturbative corrections. However, since the fermionic Matsubara frequencies do not contain 0, we cannot directly say what this implies for the solution on the real axis. The magnitude of ϕ_0 has little effect on the solution since the effect of spin and triplet fluctuations are controlled by g_N and g_d, respectively, which we keep constant.

In Fig. 4, we plot the corrections in $\varepsilon(i\omega_n)$ and $\Delta(i\omega_n)$ after including the effects of all the self energies $\Sigma = \Sigma_1 + \Sigma_2 + \Sigma_3$. We find that the inclusion Σ_1 and Σ_2 together reduces the difference between the self consistent and perturbative solution (refer to the plot near $\phi_0 \sim 0$). As we increase ϕ_0, the difference between the self consistent and

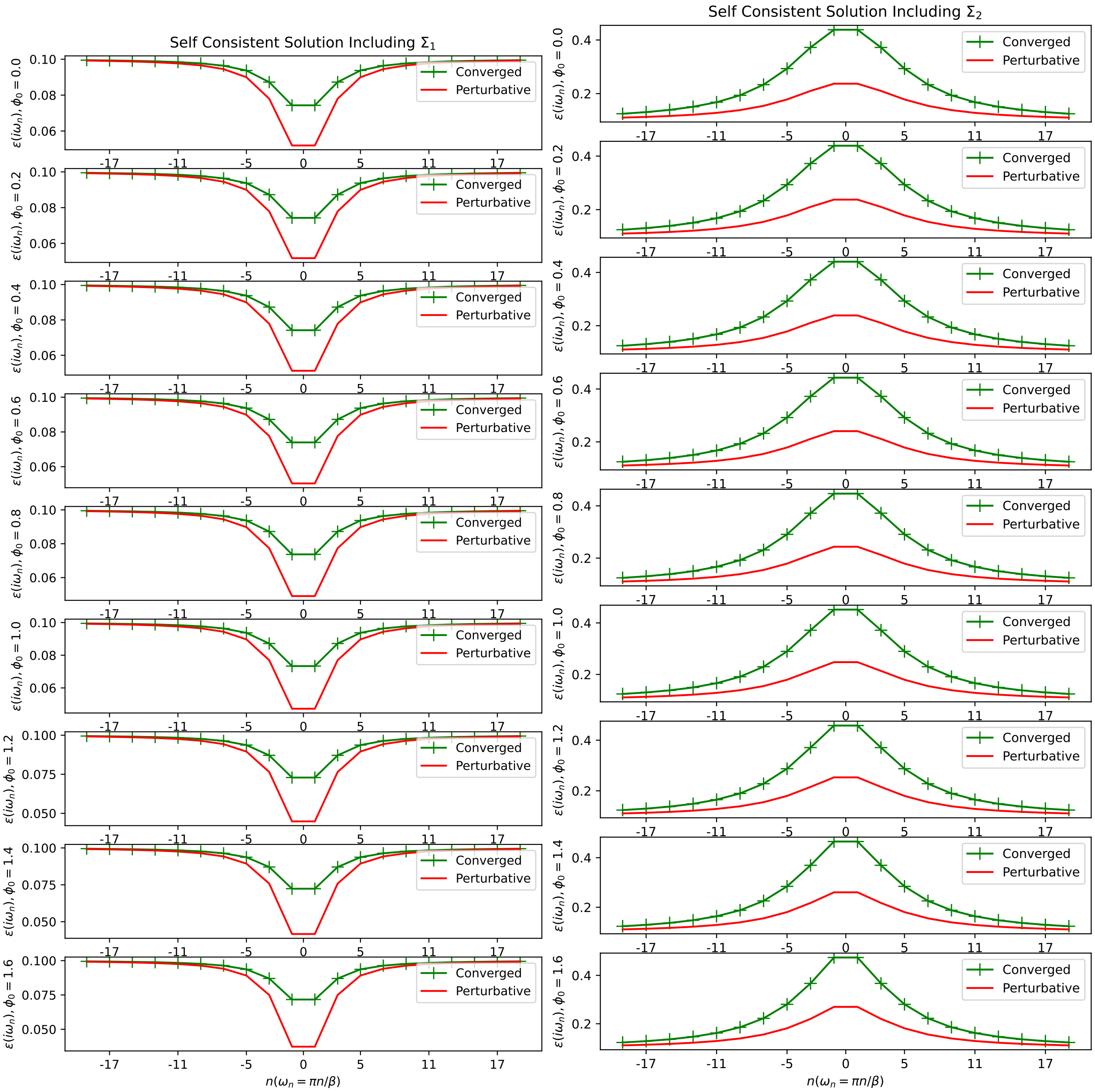

FIG. 3: The first order solution to $\varepsilon(i\omega)$ (red) and the self consistent solution (green) after including the effects of Σ_1 (left column) and Σ_2 (right column) separately. Same parameters as in Fig. 2.

perturbative solution increases due to the effect of Σ_3 which is controlled by ϕ_0.

Taken together, we see that the inclusion of second- and higher-order diagrams that contribute in the large-N limit defined in the main text yields qualitatively similar behavior on the imaginary axis compared to the first-order diagrams. We therefore expect that the qualitative picture that $\mathcal{S}_1$ renormalizes the DOS close to the Fermi level on top of which $\mathcal{S}_2$ reduces the low-energy spectral weight still applies. Since the impact of $\mathcal{S}_2$ is controlled by small ϕ_0 and good quantitative agreement is found for ϕ_0 up to $0.6r_N$, we expect that Fig. 3(c) would look similar when higher-order corrections were included.

Appendix D: Suppression of DOS at $\omega = 0$

In this section, we derive a compact approximate analytical expression for the suppression of the density of states (DOS) as a result of the anomalous term $\Sigma_3 = \Sigma_{\gamma_y}\gamma_y$. To this end, we focus on the limit of large bosonic velocities

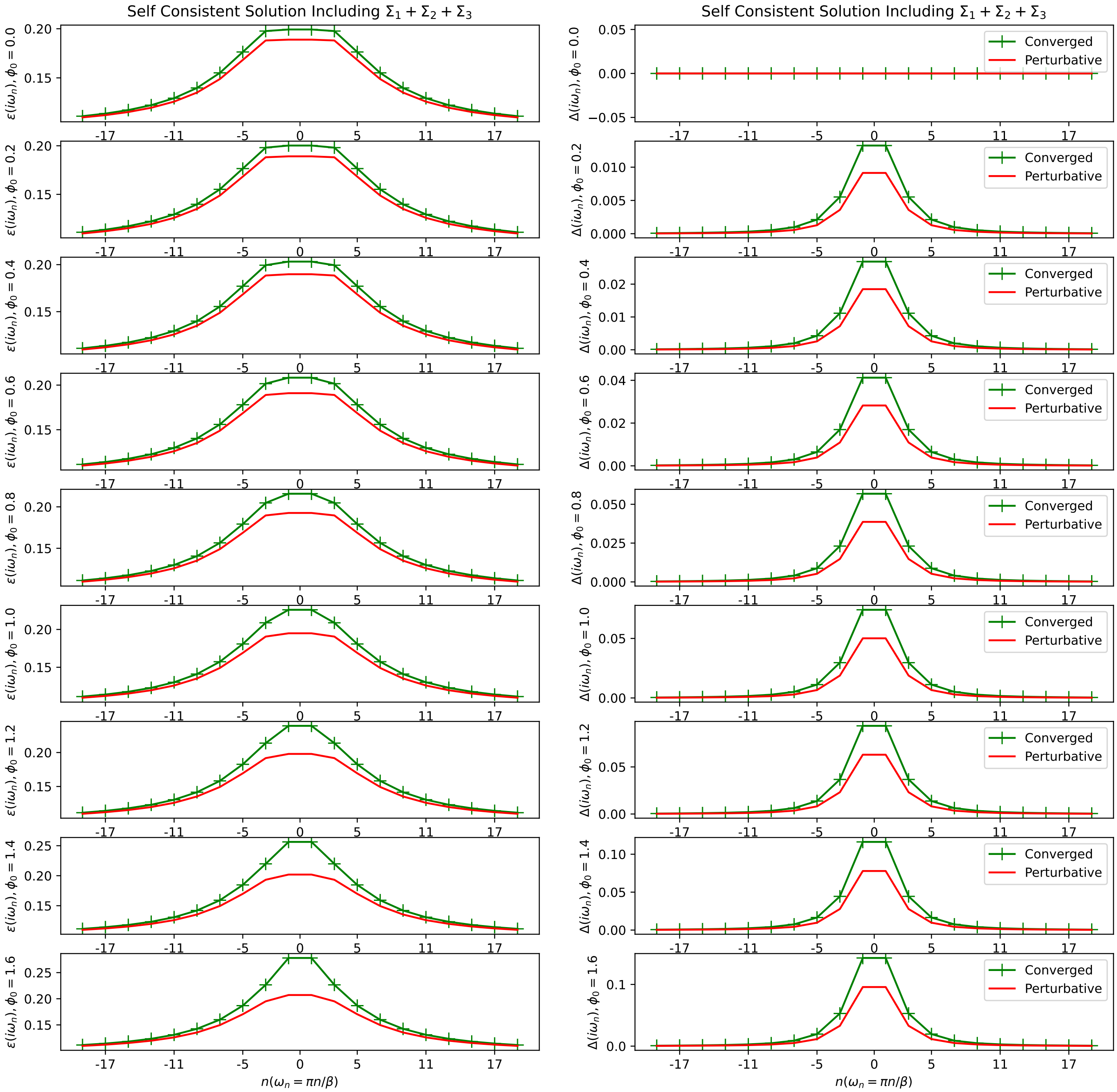

FIG. 4: The first order solution to $\varepsilon(i\omega), \Delta(i\omega)$ (red) and the self consistent solution (green) after including the effects of all the terms of the self energy $\Sigma_1 + \Sigma_2 + \Sigma_3$. Same parameters as in Fig. 2.

v_μ in χ_μ and replace the $\boldsymbol{q}$ integral in Eq. (B20) with the value of the integrand at $\boldsymbol{q} = 0$,

$$\Sigma_{\gamma_y}(\omega + i0^+, \boldsymbol{k}) = 3\phi_0 \lambda^2 \frac{r_N}{v_N^2} \left(f(\omega + i0^+, \epsilon_{\boldsymbol{k}}) - f(\omega + i0^+, -\epsilon_{\boldsymbol{k}}) \right). \tag{D1}$$

Note that we would first need to re-parametrize the integral in terms of $\tilde{\boldsymbol{q}} = \boldsymbol{q}\sqrt{r_N}/v_N$ and then set $\tilde{\boldsymbol{q}} = 0$. This approximation would then be valid in the large v_d/v_N limit with this re-scaling. We then Taylor expand $f(z, \epsilon)$ with respect to ϵ, ω, at a non-zero finite T (satisfying $\epsilon \ll T \ll \sqrt[4]{r_d r_N - \phi_0^2}$). In this limit, we find the self energy to be

$$\Sigma_{\gamma_y} = \frac{3\phi_0 r_N \lambda^2}{2 v_N^2 T (r_d r_N - \phi_0^2)} \epsilon_{\boldsymbol{k}} = \alpha \epsilon_{\boldsymbol{k}}. \tag{D2}$$

This expression is in agreement with the result in the main text [Fig. 3(a)] which shows that as $\epsilon \to 0$, the contribution of Σ_y vanishes. With such a self-energy, the spectral function is given by

$$A(\omega) = -\frac{1}{\pi}\text{Im}\,\frac{\omega + i0^+}{(\omega + i0^+)^2 - (1+\alpha^2)\epsilon_{\boldsymbol{k}}^2}. \tag{D3}$$

A simple way to look at this, is that the band structure is simply renormalized as $\epsilon_{\boldsymbol{k}} \to \sqrt{1+\alpha^2}\epsilon_{\boldsymbol{k}}$. This reduces the effective band mass, and thus the DOS is suppressed by a factor of $\sqrt{1+\alpha^2}$, as stated in the main text.

Appendix E: Demonstration of Off Diagonal Long Range Order

In this section, we calculate the ODLRO wavefunctions for both the bosons and fermions. The idea is to calculate the 4−body correlator $\langle \boldsymbol{N}(\boldsymbol{x}_1')\cdot \boldsymbol{d}(\boldsymbol{x}_2')^* \boldsymbol{N}(\boldsymbol{x}_1)\cdot \boldsymbol{d}(\boldsymbol{x}_2)\rangle$ for the bosons and $\langle c^\dagger_{\tau_1' s_1'}(\boldsymbol{x}_1') c^\dagger_{\tau_2' s_2'}(\boldsymbol{x}_2') c_{\tau_1,s_1}(\boldsymbol{x}_1) c_{\tau_2,s_2}(\boldsymbol{x}_2)\rangle$ for the fermions. Due to the $U(1)$ symmetry breaking mediated by $\boldsymbol{N}\cdot\boldsymbol{d}$ attaining a finite expectation value (and correspondingly $c^\dagger_\tau c^\dagger_{-\tau}$ for the fermions), the ODLRO factorizes into a product of functions of $\boldsymbol{x}_1 - \boldsymbol{x}_2$ and $\boldsymbol{x}_1' - \boldsymbol{x}_2'$ in the limit $\boldsymbol{x} - \boldsymbol{x}' \to \infty$, where $\boldsymbol{x} = \frac{\boldsymbol{x}_1+\boldsymbol{x}_2}{2}$ and $\boldsymbol{x}' = \frac{\boldsymbol{x}_1'+\boldsymbol{x}_2'}{2}$, giving rise to ODLRO. These wavefunctions decay as a function of their respective relative coordinates $\boldsymbol{x}_1 - \boldsymbol{x}_2$ and $\boldsymbol{x}_1' - \boldsymbol{x}_2'$. We now calculate these "macroscopic wavefunctions" explicitly for the bosonic and fermionic cases.

1. Bosonic ODLRO

The bosonic ODLRO is given by $\langle \boldsymbol{N}(\boldsymbol{x}_1')\cdot \boldsymbol{d}(\boldsymbol{x}_2')^* \boldsymbol{N}(\boldsymbol{x}_1)\cdot \boldsymbol{d}(\boldsymbol{x}_2)\rangle \simeq \langle \boldsymbol{N}(\boldsymbol{x}_1')\cdot \boldsymbol{d}(\boldsymbol{x}_2')^*\rangle\langle \boldsymbol{N}(\boldsymbol{x}_1)\cdot \boldsymbol{d}(\boldsymbol{x}_2)\rangle$ as $\boldsymbol{x}-\boldsymbol{x}' \to \infty$. All the correlators are evaluated at time $t = 0$. As discussed in the main text, to demonstrate ODLRO, it is sufficient to evaluate these correlators to first non-trivial order in the coupling constants. For bosonic ODLRO it is in fact sufficient to focus on zeroth order, i.e., neglecting the coupling to the fermions. Using the translation invariance of the system (and summing over the Matsubara frequencies $i\Omega$ since we are evaluating the correlator at time $t = 0$), we then have

$$\psi_B(\boldsymbol{x}) = \langle \boldsymbol{N}(\boldsymbol{x})\cdot \boldsymbol{d}(\boldsymbol{x}=0)\rangle = \int_{\boldsymbol{q}} T \sum_{i\Omega} e^{i\boldsymbol{q}\cdot\boldsymbol{x}} \langle \boldsymbol{N}_{-q}\cdot \boldsymbol{d}_q\rangle \tag{E1}$$

$$= \int_{\boldsymbol{q}} T \sum_{i\Omega} e^{i\boldsymbol{q}\cdot r} \frac{\phi_0}{[(i\Omega)^2 - E_+^2(\boldsymbol{q})][(i\Omega)^2 - E_-^2(\boldsymbol{q})]} \tag{E2}$$

$$= \int_{\boldsymbol{q}} e^{i\boldsymbol{q}\cdot\boldsymbol{x}} \frac{\phi_0}{2E_+(\boldsymbol{q})E_-(\boldsymbol{q})(E_+(\boldsymbol{q}) + E_-(\boldsymbol{q}))} \tag{E3}$$

$$\simeq \int_{\boldsymbol{q}} e^{i\boldsymbol{q}\cdot\boldsymbol{x}} \frac{\phi_0}{a + b\boldsymbol{q}^2} \tag{E4}$$

$$= \frac{\phi_0}{b} \int_{\boldsymbol{q}} e^{i\boldsymbol{q}\cdot\sqrt{\frac{a}{b}}\boldsymbol{x}} \frac{1}{1+\boldsymbol{q}^2} = 2\pi\phi_0 K_0\left(\sqrt{\frac{a}{b}}|\boldsymbol{x}|\right)/b \tag{E5}$$

$$= 2\pi\phi_0 K_0\left(|\boldsymbol{x}|/\xi\right)/b, \tag{E6}$$

where K_0 is the zeroth modified Bessel function of second kind. In the third line, we evaluated the Matsubara sum at $T = 0$, and in the fourth line we series expanded $2E_+(\boldsymbol{q})E_-(\boldsymbol{q})\left(E_+(\boldsymbol{q}) + E_-(\boldsymbol{q})\right)$ about $\boldsymbol{q} = 0$ up to quadratic order. The length scale $\xi = \sqrt{\frac{b}{a}}$ is determined by r_μ, v_μ. In the limit of $|v_N - v_d| \ll v_N + v_d$, we get

$$\xi = \frac{1}{2}\sqrt{\frac{(v_N^2 + v_d^2)\left(\sqrt{r_N r_d - \phi_0^2} + r_N + r_d\right)}{r_N r_d - \phi_0^2}}. \tag{E7}$$

In Fig. 5(b), we plot the numerical ODLRO wavefunction $\psi_B(\boldsymbol{x})$ with the full functional dependence on $\boldsymbol{q}$ in Eq. (E3) included, and compare it with the asymptotic analytical form in Eq. (E6). We find good agreement between the numerical and analytical results.

2. Fermionic ODLRO

Similarly, we can find the fermionic ODLRO, which in real space is generically written as $\langle c^\dagger_{\tau_1' s_1'}(\boldsymbol{x}_1')c^\dagger_{\tau_2' s_2'}(\boldsymbol{x}_2')c_{\tau_1,s_1}(\boldsymbol{x}_1)c_{\tau_2,s_2}(\boldsymbol{x}_2)\rangle \sim \langle c^\dagger_{\tau_1' s_1'}(\boldsymbol{x}_1')c^\dagger_{\tau_2' s_2'}(\boldsymbol{x}_2')\rangle\langle c_{\tau_1,s_1}(\boldsymbol{x}_1)c_{\tau_2,s_2}(\boldsymbol{x}_2)\rangle$ in the limit $\boldsymbol{x}_j - \boldsymbol{x}_j' \to \infty$. Here, τ, s are the valley and spin indices respectively. To demonstrate ODLRO, we thus have to evaluate the 2−fermion correlators, which in momentum space becomes

$$(\Psi^*_{\rm F}(\boldsymbol{x}))_{s_1,s_2} = \langle c^\dagger_{\tau_1,s_1}(\boldsymbol{x}, t=0)c^\dagger_{\tau_2,s_2}(\boldsymbol{x}=0, t=0)\rangle = \int_k e^{i\boldsymbol{k}\cdot\boldsymbol{x}}\langle c^\dagger_{k,\tau_1,s_1}c^\dagger_{-k,\tau_2,s_2}\rangle. \tag{E8}$$

Since the superconducting pairing takes place only between electrons between opposite valleys, we will have only $\tau_2 = -\tau_1$ giving non-zero correlators. Without loss of generality we chose $\tau_1 = +, \tau_2 = -$. Up to first order in ϕ_0, we have

$$\langle c^\dagger_{k,+,s_1}c^\dagger_{-k,-,s_2}\rangle = \langle c^\dagger_{k,+,s_1}c^\dagger_{-k,-,s_2}\left(-\int_q \frac{1}{2}\frac{\phi_0\lambda^2}{M_q}\mathbf{S}_q\cdot\mathbf{D}^\dagger_q\right)\rangle_0, \tag{E9}$$

where $\langle ...\rangle$ is the average with respect to the interacting and $\langle ...\rangle_0$ with respect to the non-interacting ground state. We define $G(k) = \delta_{ss'}\delta_{\tau\tau'}G_{V,k} = \frac{\delta_{ss'}\delta_{\tau\tau'}}{i\omega_n - \epsilon_{\boldsymbol{k}}} = -\langle c_{s,\tau}c^\dagger_{s',\tau'}\rangle$ to be the Green's function in the fermionic basis (assuming $\epsilon_{\boldsymbol{k}} = \epsilon_{-\boldsymbol{k}}$). Equation (E9) can then be evaluated as,

$$-\frac{\phi_0\lambda^2}{2}\int_q \frac{1}{M_q}\langle c^\dagger_{k,+,s_1}c^\dagger_{-k,-,s_2}\left(\mathbf{S}_q\cdot\mathbf{D}^\dagger_q\right)\rangle_0 \tag{E10}$$

$$= -\frac{\phi_0\lambda^2}{2}\int_q \frac{1}{M_q}\langle c^\dagger_{k+,s_1}c^\dagger_{-k-,s_2}\left(\sum_{k_1,k_2,p1=\pm,p_2=\pm} p_1p_2\left(c^\dagger_{k_1+q,p_1}\mathbf{s}c_{k_1,p_1}\right)\cdot\left(c_{k_2+q,p_2}is_y\mathbf{s}c_{-k_2,-p_2}\right)\right)\rangle_0 \tag{E11}$$

$$= -2\frac{\phi_0\lambda^2}{2}\int_q \frac{1}{M_q}\langle c^\dagger_{k+,s_1}c^\dagger_{-k-,s_2}\left(\sum_{k_1,p} -\left(c_{-k_1,-p}is_y\mathbf{s}(-G_{V,k_1+q,p})\mathbf{s}c_{k_1,p}\right)\right)\rangle_0 \tag{E12}$$

$$= -6\frac{\phi_0\lambda^2}{2}\int_q \frac{1}{M_q}\langle c^\dagger_{k+,s_1}c^\dagger_{-k-,s_2}\left(\sum_{k_1,p}\left(c_{-k_1,-p}is_yG_{V,k_1+q,p}c_{k_1,p}\right)\right)\rangle_0 \tag{E13}$$

$$= -6\frac{\phi_0\lambda^2}{2}\int_q \frac{1}{M_q}\langle c^\dagger_{k+,s_1}c^\dagger_{-k-,s_2}\left(\sum_{k_1}\left(c_{-k_1,-}is_yG_{V,k_1+q,+}c_{k_1,+} + c_{-k_1,+}is_yG_{V,k_1+q,-}c_{k_1,-}\right)\right)\rangle_0 \tag{E14}$$

$$= -6\frac{\phi_0\lambda^2}{2}\int_q \frac{1}{M_q}\left(-(-G_{V,-k,-})(is_y)_{s_2s_1}G_{V,k+q,+}G_{V,k,+} + (-G_{V,k,+})(is_y)_{s_1s_2}G_{V,-k+q,-}G_{V,-k,-}\right) \tag{E15}$$

$$= -6\frac{\phi_0\lambda^2}{2}\int_q \frac{1}{M_q}G_{V,k}G_{V,-k}\left(G_{V,-k+q} + G_{V,k+q}\right)(is_y)_{s_2s_1}. \tag{E16}$$

We continue by calculating the Matsubara sum over $i\Omega_n$ and over $i\omega_n$ [see Eq. (E8)],

$$T^2\sum_{i\omega_n,i\Omega_n}\frac{1}{(i\Omega_n^2 - E_+(\boldsymbol{q})^2)(i\Omega_n^2 - E_-(\boldsymbol{q})^2)}G_{V,k}G_{V,-k}\left(G_{V,-k+q} + G_{V,k+q}\right) \tag{E17}$$

$$= -T^2\sum_{i\omega_n,i\Omega}\frac{1}{(i\omega_n)^2 - \epsilon^2_{\boldsymbol{k}}}\frac{1}{((i\Omega_n)^2 - E_+(\boldsymbol{q})^2)((i\Omega_n)^2 - E_-(\boldsymbol{q})^2)}\left(\frac{1}{i\omega_n + i\Omega_n - \epsilon_{-\boldsymbol{k}+\boldsymbol{q}}} + \frac{1}{-i\omega_n + i\Omega_n - \epsilon_{\boldsymbol{k}+\boldsymbol{q}}}\right) \tag{E18}$$

$$=: X(\epsilon_{\boldsymbol{k}}, \boldsymbol{q}). \tag{E19}$$

For simplicity, we here focus on the limit where the remaining sum over $\boldsymbol{q}$ in Eq. (E9) is determined by its $\boldsymbol{q} = 0$ component. With $E_\pm \equiv E_\pm(\boldsymbol{q} = 0)$ and $v_N, r_N = 1$, we have

$$\hat{X}(\epsilon) \equiv X(\epsilon, \boldsymbol{q} \to 0) \tag{E20}$$

$$= \frac{n_f(\epsilon)^2}{2\epsilon}\left(-2\frac{e^{\beta\epsilon}}{E_+^2E_-^2} + \frac{2}{\left(E_+^2-4\epsilon^2\right)\left(E_-^2-4\epsilon^2\right)} + \left(\frac{2\epsilon n_B(E_+) - E_+ n_f(E_+)}{E_+(E_+^2-E_-^2)(E_+^2-4\epsilon^2)n_f(E_+)n_B(2\epsilon)} + E_+ \leftrightarrow E_-\right)\right), \tag{E21}$$

we can then finally write

$$\Psi_{\rm F}^*(\boldsymbol{x}) = 3|\phi_0|\lambda^2 s_y\left(\frac{1}{V}\sum_{\boldsymbol{k}} e^{i\boldsymbol{k}\cdot\boldsymbol{x}}\hat{X}(\epsilon_{\boldsymbol{k}})\right), \tag{E22}$$

$$= \frac{3|\phi_0|\lambda^2 s_y}{2\pi}\int_0^\infty dkkJ_0(\boldsymbol{k}\cdot\boldsymbol{x})\hat{X}(\hbar^2(\boldsymbol{k}^2-\boldsymbol{k}_F^2)/(2m)). \tag{E23}$$

In the second line, we assumed $\epsilon_{\boldsymbol{k}} = \hbar^2(\boldsymbol{k}^2-\boldsymbol{k}_F^2)/2m$. Using this expression, we calculate the spatial profile of the fermionic ODLRO wavefunction numerically for various values of $\epsilon_F \equiv \epsilon_{\boldsymbol{k}_F}$ in Fig. 5(a). Unlike the case of the bosonic ODLRO (which was exponentially decaying), the fermionic ODLRO has an oscillating component superimposed on an exponentially decaying envelope.

Appendix F: Meissner Effect From ODLRO

The consequences of ODLRO defined in terms of four-fermion or two-boson correlators are well-known [1–4]. As a result of spin-rotation symmetry, we cannot capture ODLRO using a correlator of only two bosons. Instead, we have to study the four-boson density matrix

$$\rho(\boldsymbol{x}_1,\boldsymbol{x}_2,\boldsymbol{x}_1',\boldsymbol{x}_2') = \langle \boldsymbol{N}(\boldsymbol{x}_1)\cdot\boldsymbol{d}^*(\boldsymbol{x}_2)\boldsymbol{N}(\boldsymbol{x}_1')\cdot\boldsymbol{d}(\boldsymbol{x}_2')\rangle. \tag{F1}$$

Although the derivation is in close analogy to the two-boson or four-fermion case, we here show explicitly how the Meissner effect follows from

$$\rho(\boldsymbol{x}_1,\boldsymbol{x}_2,\boldsymbol{x}_1',\boldsymbol{x}_2') \to \phi_0^*(\boldsymbol{x}_1,\boldsymbol{x}_2)\phi_0(\boldsymbol{x}_1',\boldsymbol{x}_2') \neq 0, \quad |\boldsymbol{x}_j-\boldsymbol{x}_j'|\to\infty. \tag{F2}$$

Let us consider the system to be in the presence of a spatially uniform orbital magnetic field of strength $\boldsymbol{B} = B_0\hat{z}$ in the out of plane direction. Note that an in-plane orbital magnetic field does not couple to the bosons as the spatial motion is constrained to the two-dimensional plane of the system. The corresponding vector potential is given by $\boldsymbol{A}(\boldsymbol{x}) = \frac{1}{2}\boldsymbol{B}\times\boldsymbol{x}$, with $\boldsymbol{x} = (x,y,0)$. Under an in-plane translation by $\boldsymbol{a}$, the vector potential transforms as

$$\boldsymbol{A}(\boldsymbol{x}) \to \boldsymbol{A}(\boldsymbol{x}-\boldsymbol{a}) = \boldsymbol{A}(\boldsymbol{x}) - \frac{1}{2}\boldsymbol{B}\times\boldsymbol{a} \tag{F3}$$

$$= \boldsymbol{A}(\boldsymbol{x}) - \frac{1}{2}\nabla\left[\boldsymbol{a}\cdot(\boldsymbol{x}\times\boldsymbol{B})\right] \tag{F4}$$

$$= \boldsymbol{A}(\boldsymbol{x}) + \nabla\chi_{\boldsymbol{a}}(\boldsymbol{x}), \tag{F5}$$

where $\chi_{\boldsymbol{a}}(\boldsymbol{x}) = -\frac{1}{2}\boldsymbol{a}\cdot(\boldsymbol{x}\times\boldsymbol{B})$. Note that the triplet pairing field $\boldsymbol{d}$ is a charge-$2e$ bosonic field, while the magnetization field $\boldsymbol{N}$ is neutral. Therefore, under simultaneous gauge transformation and displacement by $\boldsymbol{a}$ in the presence of a magnetic field, the fields transform as

$$\boldsymbol{d}(\boldsymbol{x}) \to e^{i\frac{2e}{\hbar c}\chi_a(\boldsymbol{x})}\boldsymbol{d}(\boldsymbol{x}-\boldsymbol{a}), \tag{F6}$$

$$\boldsymbol{N}(\boldsymbol{x}) \to \boldsymbol{N}(\boldsymbol{x}-\boldsymbol{a}), \tag{F7}$$

$$\boldsymbol{A}(\boldsymbol{x}) \to \boldsymbol{A}(\boldsymbol{x}). \tag{F8}$$

As a result of gauge covariance and translational symmetry, the four-body density matrix obeys

$$\rho(\boldsymbol{x}_1,\boldsymbol{x}_2,\boldsymbol{x}_1',\boldsymbol{x}_2') = e^{i\frac{2e}{\hbar c}\left(\chi_{\boldsymbol{a}}(\boldsymbol{x}_2')-\chi_{\boldsymbol{a}}(\boldsymbol{x}_2)\right)}\rho(\boldsymbol{x}_1-\boldsymbol{a},\boldsymbol{x}_2-\boldsymbol{a},\boldsymbol{x}_1'-\boldsymbol{a},\boldsymbol{x}_2'-\boldsymbol{a}). \tag{F9}$$

Now suppose the system has ODLRO, i.e., Eq. (F2) holds. In combination with Eq. (F9), this implies

$$\phi_0^*(\boldsymbol{x}_1,\boldsymbol{x}_2)\phi_0(\boldsymbol{x}_1',\boldsymbol{x}_2') = e^{i\frac{2e}{\hbar c}\left(\chi_{\boldsymbol{a}}(\boldsymbol{x}_2')-\chi_{\boldsymbol{a}}(\boldsymbol{x}_2)\right)}\phi_0^*(\boldsymbol{x}_1-\boldsymbol{a},\boldsymbol{x}_2-\boldsymbol{a})\phi_0(\boldsymbol{x}_1'-\boldsymbol{a},\boldsymbol{x}_2'-\boldsymbol{a}) \tag{F10}$$

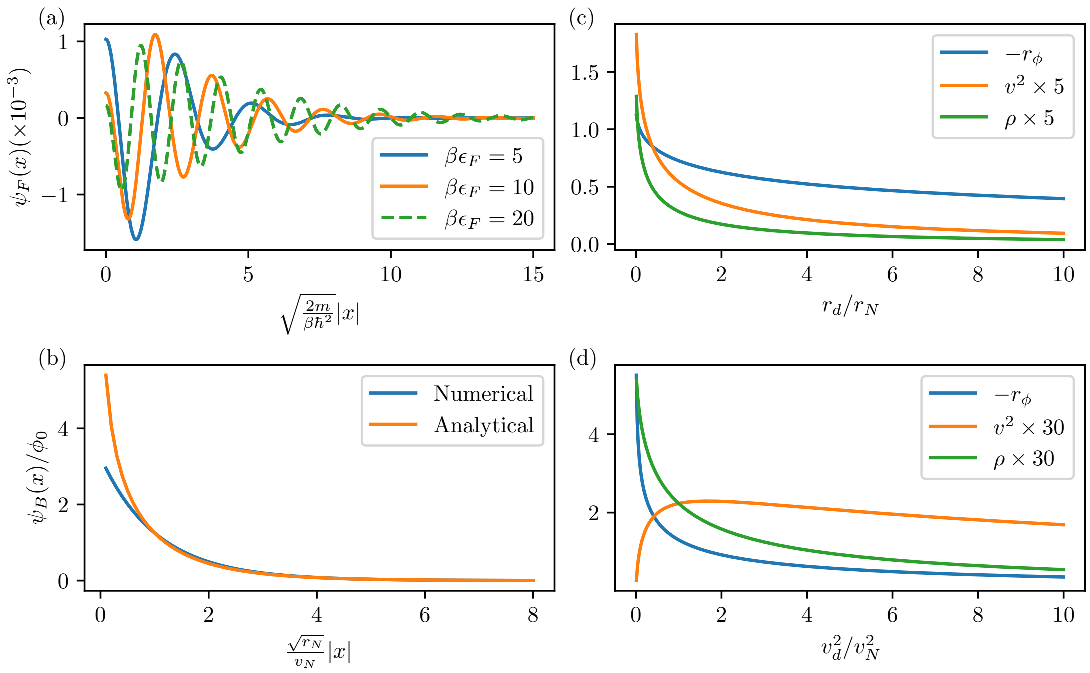

FIG. 5: (a) The fermionic and (b) the bosonic ODLRO "macroscopic wavefunction". The mass r_ϕ [in units of $r_N^{-1/2} v_N^{-2}$], superfluid density ρ [$r_N^{-3/2} v_N^{-2}$], and velocity v^2 [$r_N^{-3/2}$] of $\mathcal{S}_{\rm GL}$ in Eq. (G17) as a function of r_d and v_d^2 are shown in (c) and (d), respectively.

$$\implies \phi_0(\boldsymbol{x}_1, \boldsymbol{x}_2) = f_{\boldsymbol{a}} e^{i\frac{2e}{\hbar c}\chi_{\boldsymbol{a}}(\boldsymbol{x}_2)} \phi_0(\boldsymbol{x}_1 - \boldsymbol{a}, \boldsymbol{x}_2 - \boldsymbol{a}), \tag{F11}$$

where $f_{\boldsymbol{a}}$ is a position-independent phase factor. Now suppose we perform two different translations by $\boldsymbol{a}$ and $\boldsymbol{b}$. We can perform $\boldsymbol{a}$ first and then $\boldsymbol{b}$. Alternatively, we can do $\boldsymbol{b}$ first and then $\boldsymbol{a}$. They respectively give us

$$\phi_0(\boldsymbol{x}_1, \boldsymbol{x}_2) = f_{\boldsymbol{b}} f_{\boldsymbol{a}} e^{i\frac{2e}{\hbar c}\chi_{\boldsymbol{a}}(\boldsymbol{x}_2)} e^{i\frac{2e}{\hbar c}\chi_{\boldsymbol{b}}(\boldsymbol{x}_2 - \boldsymbol{a})} \phi_0(\boldsymbol{x}_1 - \boldsymbol{a}, \boldsymbol{x}_2 - \boldsymbol{a}), \tag{F12}$$

$$\phi_0(\boldsymbol{x}_1, \boldsymbol{x}_2) = f_{\boldsymbol{b}} f_{\boldsymbol{a}} e^{i\frac{2e}{\hbar c}\chi_{\boldsymbol{b}}(\boldsymbol{x}_2)} e^{i\frac{2e}{\hbar c}\chi_{\boldsymbol{a}}(\boldsymbol{x}_2 - \boldsymbol{b})} \phi_0(\boldsymbol{x}_1 - \boldsymbol{a}, \boldsymbol{x}_2 - \boldsymbol{a}). \tag{F13}$$

This is only consistent if

$$e^{i\frac{2e}{\hbar c}(\chi_{\boldsymbol{b}}(\boldsymbol{x}_2) + \chi_{\boldsymbol{a}}(\boldsymbol{x}_2 - \boldsymbol{b}) - \chi_{\boldsymbol{a}}(\boldsymbol{x}_2) - \chi_{\boldsymbol{b}}(\boldsymbol{x}_2 - \boldsymbol{a}))} = 1. \tag{F14}$$

We can evaluate $\chi_{\boldsymbol{b}}(\boldsymbol{x}_2) + \chi_{\boldsymbol{a}}(\boldsymbol{x}_2 - \boldsymbol{b}) - \chi_{\boldsymbol{a}}(\boldsymbol{x}_2) - \chi_{\boldsymbol{b}}(\boldsymbol{x}_2 - \boldsymbol{a}) = \boldsymbol{B} \cdot (\boldsymbol{a} \times \boldsymbol{b})$. Thus, the condition for equality of phases becomes

$$\frac{2e}{\hbar c} \boldsymbol{B} \cdot (\boldsymbol{a} \times \boldsymbol{b}) = 2\pi n, \tag{F15}$$

for some integer n. The only solution for arbitrary $\boldsymbol{a}, \boldsymbol{b}$ is thus $\boldsymbol{B} = 0$.

Appendix G: Ginzburg-Landau theory

We here calculate the Landau-Ginzburg theory for the bosonic superfluid condensate parameter to leading (zeroth) order in the fermion-boson coupling λ. To tis end, we assume that ϕ_0 is now spatially and temporally varying. This results in non-zero Fourier modes ϕ_q for $\boldsymbol{q}, i\Omega \neq 0$.

In momentum space, the bosonic action is generalized according to

$$\mathcal{S}_B = \int_q [\chi_N^{-1}(q) \boldsymbol{N}_q \cdot \boldsymbol{N}_{-q} + \chi_{SC}^{-1}(q) \boldsymbol{d}_q^* \cdot \boldsymbol{d}_q + (\phi_0 \boldsymbol{d}_q \cdot \boldsymbol{N}_{-q} + \text{H.c.})] \tag{G1}$$

$$= \int_q \begin{pmatrix} \boldsymbol{N}_{-q}^T & \boldsymbol{d}_q^\dagger \end{pmatrix} \begin{pmatrix} \chi_N^{-1}(q) & \phi_0 \\ \phi_0 & \chi_d^{-1}(q) \end{pmatrix} \begin{pmatrix} \boldsymbol{N}_q \\ \boldsymbol{d}_q \end{pmatrix} \tag{G2}$$

$$\to \int_{q,k} \begin{pmatrix} \boldsymbol{N}^T_{-q-q_2} & \boldsymbol{d}^\dagger_{q+q_2} \end{pmatrix} \begin{pmatrix} \chi_N^{-1}(q)\delta_{q_2=0} & \phi_{q_2} \\ \phi^*_{-q_2} & \chi_d^{-1}(q)\delta_{q_2=0} \end{pmatrix} \begin{pmatrix} \boldsymbol{N}_q \\ \boldsymbol{d}_q \end{pmatrix} \tag{G3}$$

So after integrating out $\boldsymbol{d}$ and $\boldsymbol{N}$, the effective action for ϕ reads as

$$\mathcal{S}_{\text{eff}} = \frac{1}{2} Tr \ln G^{-1}[\phi], \tag{G4}$$

where

$$G^{-1}[\phi](q+q_1,q) = G_0^{-1}(q)\delta_{q_1,0} + \Gamma_{q+q_1,q} \tag{G5}$$

$$G_0^{-1} = \begin{pmatrix} \chi_N^{-1}(q) & 0 \\ 0 & \chi_d^{-1}(q) \end{pmatrix} \tag{G6}$$

$$\Gamma_{q+q_1,q} = \begin{pmatrix} 0 & \phi_{q_1} \\ \phi^*_{-q_1} & 0 \end{pmatrix}. \tag{G7}$$

To derive the Ginzburg-Landau theory for ϕ, we expand $Tr \ln G^{-1}$ upto second order in Γ, which is equivalent to second order in ϕ. This gives us

$$S_{\text{GL}} = Tr \ln(G_0^{-1} + \Gamma) \simeq Tr G_0^{-1} + Tr G_0 \Gamma - \frac{1}{2} Tr G_0 \Gamma G_0 \Gamma \tag{G8}$$

Because of the diagonal structure of G_0, and the off diagonal structure of Γ, the linear term $Tr G_0 \Gamma$ is 0. The quadratic term becomes

$$\sum_{q',q} Tr G_0(q'+q)\Gamma(q'+q,q')G_0(q')\Gamma(q',q'+q) = \sum_{q',q} Tr \begin{pmatrix} 0 & \chi_N(q'+q)\phi_q \\ \chi_d(q'+q)\phi^*_{-q} & 0 \end{pmatrix} \begin{pmatrix} 0 & \chi_N(q')\phi_{-q} \\ \chi_d(q')\phi^*_q & 0 \end{pmatrix} \tag{G9}$$

$$= \sum_{q',q} \chi_N(q'+q)\chi_d(q')\phi_q\phi^*_q + \chi_N(q')\chi_d(q'+q)\phi_{-q}\phi^*_{-q} \tag{G10}$$

$$= \sum_{q',q} \left(\chi_N(q'+q)\chi_d(q') + \chi_N(q')\chi_d(q'-q)\right)\phi_q\phi^*_q \tag{G11}$$

$$= \sum_{q',q} \left(\chi_N(q'+q)\chi_d(q') + \chi_N(q'+q)\chi_d(q')\right)\phi_q\phi^*_q \tag{G12}$$

$$= 2\sum_{q',q} \chi_N(q'+q)\chi_d(q')\phi_q\phi^*_q \tag{G13}$$

We need to evaluate

$$\sum_{q'} \chi_N(q'+q)\chi_d(q') = \int_{\boldsymbol{q}'} T \sum_{i\Omega' \in \text{Bosonic}} \left(\frac{1}{((i\Omega'+i\Omega)^2 - r_N - v_N^2(\boldsymbol{q}'+\boldsymbol{q})^2)((i\Omega')^2 - r_d - v_d^2\boldsymbol{q}'^2)} \right) \tag{G14}$$

$$= -\frac{1}{2}\int_{\boldsymbol{q}'} \left(\frac{1}{\sqrt{r_N + v_N^2(\boldsymbol{q}'+\boldsymbol{q}/2)^2}} + \frac{1}{\sqrt{r_d + v_d^2(\boldsymbol{q}'-\boldsymbol{q}/2)^2}} \right) \left(\frac{1}{i\Omega^2 - \left(\sqrt{r_N + v_N^2(\boldsymbol{q}'+\boldsymbol{q}/2)^2} + \sqrt{r_d + v_d^2(\boldsymbol{q}'-\boldsymbol{q}/2)^2}\right)^2} \right). \tag{G15}$$

By expanding the above expression up to second order in $i\Omega$, $\boldsymbol{q}$, we find the effective action for the ϕ field to be

$$\mathcal{S}_{\text{GL}} = \int_{\boldsymbol{x},\tau} \left[\rho|D_\tau\phi|^2 + r_\phi|\phi|^2 + v^2\left|\boldsymbol{D}\phi\right|^2\right] \tag{G16}$$

$$= T\sum_{i\Omega,\boldsymbol{q}} \left(r_\phi - \rho(i\Omega)^2 + v^2\boldsymbol{q}^2\right)|\phi_{(\boldsymbol{q},i\Omega)}|^2 \tag{G17}$$

where the coefficients are given by

$$r_\phi = -\int_{\boldsymbol{q}'} \frac{\pi}{\sqrt{g_d}\sqrt{g_N}\left(\sqrt{g_d}+\sqrt{g_N}\right)} \tag{G18}$$

$$\rho = \int_{\boldsymbol{q}'} \frac{\pi}{\sqrt{g_d}\sqrt{g_N}\left(\sqrt{g_d}+\sqrt{g_N}\right)^3} \tag{G19}$$

$$v^2 = \int_{\boldsymbol{q}'} \frac{\pi\left(4\boldsymbol{q}'^2(\sqrt{g_d}+\sqrt{g_N})\left(\frac{v_d^2}{g_d^{3/2}}-\frac{v_N^2}{g_N^{3/2}}\right)\left(\frac{v_N^2}{\sqrt{g_N}}-\frac{v_d^2}{\sqrt{g_d}}\right)-\left(\sqrt{g_d}+\sqrt{g_N}\right)^2\left(\frac{v_d^2\left(3v_d^2\boldsymbol{q}'^2-2g_d\right)}{g_d^{5/2}}+\frac{v_N^2\left(3v_N^2\boldsymbol{q}'^2-2g_N\right)}{g_N^{5/2}}\right)\right)}{16\left(\sqrt{g_d}+\sqrt{g_N}\right)^4} \tag{G20}$$

$$-\frac{2\pi\left(\frac{1}{\sqrt{g_d}}+\frac{1}{\sqrt{g_N}}\right)\left(\frac{3\boldsymbol{q}'^2\left(\sqrt{g_N}v_d^2-\sqrt{g_d}v_N^2\right)^2}{g_d g_N}-\left(\sqrt{g_d}+\sqrt{g_N}\right)\left(\frac{v_d^2\left(2g_d-\boldsymbol{q}'^2v_d^2\right)}{g_d^{3/2}}+\frac{v_N^2\left(2g_N-\boldsymbol{q}'^2v_N^2\right)}{g_N^{3/2}}\right)\right)}{16\left(\sqrt{g_d}+\sqrt{g_N}\right)^4} \tag{G21}$$

with $g_\mu = r_\mu + v_\mu^2\boldsymbol{q}'^2$. We numerically calculate the quantities r_ϕ, ρ, v^2 and plot it in Fig. 5(c,d).

[1] C. N. Yang, "Concept of Off-Diagonal Long-Range Order and the Quantum Phases of Liquid He and of Superconductors," Reviews of Modern Physics **34**, 694 (1962).

[2] G. L. Sewell, "Off-diagonal long-range order and the Meissner effect," Journal of Statistical Physics **61**, 415 (1990).

[3] H. T. Nieh, G. Su, and B.-H. Zhao, "Off-diagonal long-range order: Meissner effect and flux quantization," Physical Review B **51**, 3760 (1995).

[4] G. L. Sewell, "Off-diagonal long range order and superconductive electrodynamics," Journal of Mathematical Physics **38**, 2053 (1997).
